# Frontiers and hotspots of high-intensity interval exercise in children and adolescents: text mining and knowledge domain visualization

**DOI:** 10.3389/fphys.2024.1330578

**Published:** 2024-03-06

**Authors:** Fucheng Sun

**Affiliations:** Department of Physical Education, Faculty of Social Science, Nanjing Agricultural University, Nanjing, China

**Keywords:** interval training, child health, CiteSpace, collaboration network analysis, spatial clustering, bibliometric analysis, visualization mapping

## Abstract

**Background:** During the past two decades, research on high-intensity interval exercise (HIIE) in children and adolescents has steadily accumulated, especially on the subthemes of improving cardiometabolic and cardiovascular health. However, there is still little scientific understanding of using scientometric analysis to establish knowledge maps. Exploring the relationship between known and new emerging ideas and their potential value has theoretical and practical implications in the context of a researcher’s limited ability to read, analyze, and synthesize all published works.

**Objective:** First, this study aims to provide extensive information on HIIE research in children and adolescents, including authors, institutions, countries, journals, and references. Second, the objective is to use co-occurrence, burst, and co-citation analyses based on hybrid node types to reveal hotspots and forecast frontiers for HIIE research in children and adolescents.

**Methods:** Using the bibliographic data of the Web of Science Core Collection (WoSCC) as the data source, publications, authors, and journals were analyzed with the help of bibliometric methods and visualization tools such as CiteSpace, VOSviewer, Pajek, and Bibliometrix R package. Authorial, institutional, and national collaboration networks were plotted, along with research hotspots and research frontiers based on keyword bursts and document co-citations.

**Results:** This study found that executive function, high-intensity interval training, heart rate variability, and insulin resistance are emerging research topics; high-intensity training, mental health, exercise intensity, and cardiometabolic risk factors are continual frontier research areas in the subthemes.

**Conclusion:** Our study has three novel contributions. First, it explicitly and directly reflects the research history and current situation of the HIIE intervention strategy in children and adolescents. This approach makes it clear and easy to trace the origin and development of this strategy in specific groups of children and adolescents. Second, it analyzes the research hotspots of HIIE in the field and predicts the research frontiers and development trends, which will help researchers get a deeper understanding of HIIE and pediatric health research. Third, the findings will enable researchers to pinpoint the most influential scholars, institutions, journals, and references in the field, increasing the possibility of future collaborations between authors, institutions, and countries.

## Introduction

The idea of high-intensity interval exercise (HIIE) could be traced back to the practice of some famous athletes; e.g., in the 1920s, Olympic champion Paavo Nurmi tried to improve middle- and long-distance running performance through sprint training, and his innovative training method was the earliest exploration of HIIE. However, in the 1950s, Olympic distance running champion Emil Zatopek improved on this foundation; he used 60 × 400 m interval training so that HIIE became the standard in the field of middle-distance running and became popular among athletes (Billat, 2001). Athletes’ unwitting training practices were shown to be correct years later, a process that could not have been accomplished without the extensive contributions of physiologists. There is no uniform definition of HIIE because HIIE studies vary in intensity, dose, and manner for different purposes (Bond et al., 2017). However, physiologists generally agree that HIIE refers to a method that involves repeated short-to-long bouts of high-intensity exercise interspersed with recovery periods, often performed with an “all-out” effort or at an intensity close to peak VO_2_ (Gibala and McGee, 2008; Costigan, 2015; Gillen and Gibala, 2018). As HIIE is often promoted as a time-saving and fun exercise intervention strategy, it usually does not require expensive specialized equipment or facilities. Furthermore, HIIE is applicable for promoting cardiorespiratory fitness and modifying the prevention of cardiometabolic and cardiovascular disease risk factors. This makes it a health promotion strategy advocated by many physiologists, including some researchers who are rigorous (Gillen and Gibala, 2014) and critical thinkers (Biddle and Batterham, 2015) about the feasibility of HIIE. However, physiologists generally agree that further research on HIIE is required to explore its full potential (Gray, 2016; Eddolls, 2017; Sabag, 2022).

Comparatively, HIIE has been investigated across several experimental studies to promote athletic performance (Buchheit and Laursen, 2013), fat loss (Khalafi and Symonds, 2021), aerobic fitness (Rowan, Kueffner and Stavrianeas, 2012), and cardiometabolic risk factors in adults (Batacan, 2017). In addition, it has been generally concluded that HIIE is safe and effective. Considering the World Health Organization’s recommendation that children (defined by the Convention on the Rights of the Child as every human being below the age of 18 years) and adolescents (defined by the World Health Organization as the second decade of life, i.e., of the age of 10 to 19 years) engage in at least 60 min of moderate-to-vigorous physical activity per day to prevent potential cardiovascular disease and risk in adulthood and old age, few children and adolescents meet such guidelines (Townsend, 2015). So, in the context of physical inactivity in children and adolescents and an increasing burden of chronic diseases, researchers consider the feasibility of HIIE to be able to redress the risk of inactive lifestyles (Logan, 2014). Early studies on children and adolescents were rigorous due to participants’ parental agreement and ethical considerations. Prior to the HIIE observational study under natural field conditions, it was found that children are accustomed to short and vigorous physical activity, usually lasting no more than 15 s, interspersed with varying degrees of low-to-moderate-intensity recovery activities (Bailey et al., 1995). Baquet (2007) obtained similar conclusions when using the high-frequency acceleration method to monitor habitual physical activity in prepubertal children, arguing that regardless of intensity, children’s physical activity patterns are highly transient and intermittent. Subsequent studies have gradually begun to track the cardiorespiratory and cardiovascular health benefits of HIIE interventions and have drawn generally beneficial conclusions (Carson et al., 2014; Cockcroft et al., 2018; Duncombe et al., 2022; , 2021; Weston et al., 2016).

Regarding the reviews of this subtheme, some studies have evaluated the health benefits of HIIE on children and adolescents, discussed the limitations of HIIE research, and proposed future research. For example, Costigan et al. (2015) evaluated the effectiveness of HIIE in improving adolescents’ health adaptability. They concluded that HIIE had a large effect on cardiorespiratory fitness (d = 1.05; 95% CI: 0.36–1.75) and a medium effect on body mass index (BMI) (d = −0.37, 95% CI: −0.68 to −0.05) and body fat percentage (d = −0.67; 95% CI: −1.30 to −0.04). They also found that study duration (≥8 weeks) was a significant moderator (MD = −2.1%; 95% CI: −3.3 to −0.8; *p* = 0.001) for the effect of HIIE on body fat. Future research is encouraged to assess the associated benefits of embedding HIIE within the school day. Eddolls et al. (2017) reviewed the effects of HIIE on crucial health parameters in children and adolescents and found that HIIE was an effective method to improve biomarkers of cardiovascular disease. However, evidence for other health-related outcomes, such as body composition and blood pressure, remains unclear and equivocal. Future research should report broader perspectives on extraneous factors, such as gender and maturity differences. Bond et al. (2017) outlined the health benefits of HIIE in children and adolescents’ health promotion, obesity management, and the unique challenges encountered in school-based training interventions. However, further work is needed to optimize the delivery of HIIE interventions to improve participant enjoyment and acceptability. Recently, You et al. (2021) used bibliometric analysis and visualization technology to explore the hotspots and frontiers of HIIE in the health promotion domain and also came to encouraging conclusions. They summarized “metabolic diseases,” “cardiovascular diseases,” “neurological diseases,” and “musculoskeletal diseases” as research hotspots and “prevention and rehabilitation,” “micro and molecular level,” and “cognition and mental health” as frontier research topics. This research offered both a historical and a prospective insight into the HIIE strategy. Overall, Costigan et al. (2015); Bert Bond et al. (2017); and Eddolls et al. (2017) focused on examining the health benefits of HIIE in children and adolescents, i.e., improving blood glucose, blood lipids, aerobic fitness, and cardiovascular risk factors, while contributing data on combined effects and potential moderators. However, due to the different foci of researchers, diversity in study designs, and sample size limitations, the meta-analysis and narrative review have limited ability to draw conclusions, which poses challenges for other researchers in examining the hotspots and frontiers of HIIE. You et al. (2021) pioneered the use of the bibliometric strategy in the investigation of the HIIE health promotion domain. Their study is helpful to researchers in extracting hidden information for further studies in exercise and health-related fields and provides valuable insights. However, the data retrieved in this study did not take into account the subject factors, lacking observations of children and adolescents. Considering that active lifestyle and habits are often developed very early in childhood and adolescence, they can lead to youth and adulthood (Telama et al., 2014). This reminds researchers to pay more attention to children and adolescents and detect more suitable exercise intervention protocols for this cohort. As a whole, the increased publication volume of any discipline is much higher than the researcher’s reading, analysis, and synthesis abilities. It would also be insightful to explore the relationship between known and new ideas and discover early signs and their potential value by replacing tedious retrieval and analyses with bibliometric and visualized analysis. Importantly, this data-based description avoids subjective judgments so that it may reveal a different structure of the field than traditional literature reviews.

This study primarily analyzes collaboration networks of research on HIIE in children and adolescents, as well as research hotspots and research frontiers based on keyword burst and document co-citations. The specific issues discussed in this paper are (1) to understand the changes in the number of publications and citations of HIIE in children and adolescents, as well as the clustering and cooperative relationships among countries, institutions, and authors; (2) using keyword timeline clustering and keyword burst analysis to study the development context and emerging topics of HIIE in children and adolescents; (3) and identifying network clusters in document co-citation analysis and analyzing research frontiers in co-citation document mapping.

## Materials and methods

### Data collection and preprocessing

The scientific nature of the knowledge map relies on reliable and accurate data and the retrieval of all literature reports on research topics as much as possible. This big data paradigm avoids sampling errors to some extent. At the same time, it is imperative to choose a database that is recognized by the scientific community, has authority, and has an extensive dataset of documents. The papers included in the Web of Science (WoS) have been peer-reviewed. Compared with Scopus and Google Scholar, WoS performed the best with total coverage of the journal sample population and retrieved the most unique items (Adriaanse et al., 2013). Furthermore, the complete and consistent format of cited references in WoS greatly expedited the processing stage when generating networks to identify highly recognized documents that may contain noteworthy concept symbols, which is more suitable for mining and visual analysis of scientific and technological texts (Trujillo and Long, 2018). The search strategy constructed in this study and the steps to screen the qualified literature were as follows: first, all research data were retrieved from the Science Citation Index Expanded (SCI-EXPANDED) of the Web of Science Core Collection database on 15 November 2022. The retrieval was completed within the same day to avoid any bias caused by database updates. The Boolean logic used for the search was TS = [(“HIIT” OR “HIIE” OR “high intensity interval exercise*” OR “high intensity interval train*” OR “high intensity interval activity*” OR “high intensity intermittent exercise*” OR “high intensity intermittent train*” OR “high intensity intermittent activity*” OR “sprint interval exercise*” OR “sprint interval train*” OR “aerobic interval exercise*” OR “aerobic interval train*” OR “repeated sprint training”) AND (“adolescent*” OR “child*” OR “teen*” OR “youth” OR “boy*” OR “girl*” OR “student*”)]; the search period was set to 2002–2022, with the document type selected as Article and Review and language selected as English. A total of 399 documents were retrieved after assembly and saved as plain text. Second, the delete duplicate records function in CiteSpace is used to delete duplicate documents. The third procedure was to use Bibliometrix, VOSviewer, Pajek, and CiteSpace for scientometric analysis of the 399 eligible literature records that were finally included in this review.

### Bibliometrix, VOSviewer, CiteSpace, and Pajek overview and main functions

Scientometrics is a discipline that applies mathematical statistics and computational techniques to measure and analyze research processes, research networks, and development trends (Hood and Wilson, 2001). In recent years, with the development of visualization technology, it has become possible to transform abstract information and data in the scientific and technical literature into a visualized spatial structure and present more comprehensive information (Chen, 2006). However, bibliometric analysis software has its limitations, and no single software program currently addresses the entire workflow. Bibliometrix, VOSviewer, CiteSpace, and Pajek were the four chosen analysis software packages for this review. Bibliometrix is an open-source tool programmed in R; VOSviewer and CiteSpace are visual analysis software programs running in Java; and Pajek is an analytical tool for large networks with efficient algorithms. This study uses Bibliometrix to provide a basic description of the data and a visual analysis of inflows and outflows between authors, affiliations, and sources. VOSviewer and CiteSpace carry out collaborative network analysis, and Pajek optimizes the spatial layout of the visualization network. CiteSpace performs co-occurrence analysis, burst detection, and document co-citation analysis. Bibliometrics software such as Bibliometrix, VOSviewer, CiteSpace, and Pajek focus on analyzing the latent knowledge contained in the scientific literature and presenting it visually; among them, CiteSpace has advantages in detecting the structural centrality in node networks and mapping research frontiers (Li and Chen, 2017). The implementation process is to detect and monitor the evolution of a knowledge domain. Many aspects of a knowledge domain can be represented in different forms of scientific networks. The method first derives a sequence of co-citation networks from a series of equal-length time slices. These time-registered networks are merged and visualized in a panoramic view where the visually salient nodes can be identified based on their features. The most important aspect is that the search for intellectual turning points can be narrowed down to visually salient nodes in the visualized network. Therefore, the representative literature among the turning points and the evolution process of the field can be analyzed. The designer of CiteSpace regards the citation relationship between documents based on a unique perspective: whether someone supports or opposes the previous point of view, it is apparent that the researchers cannot ignore its existence, and it becomes part of this paradigm (Chen, 2004). In summary, bibliometrics provides a more in-depth and objective content analysis based on data. However, bibliometrics is not a complete substitute for extensive reading in the field as scholars with in-depth knowledge also have distinct advantages. Future studies should fuse two methods to facilitate the presentation of high-level results.

## Results and discussion

### General characteristics of the publications on HIIE in children and adolescents

**FIGURE 1:**
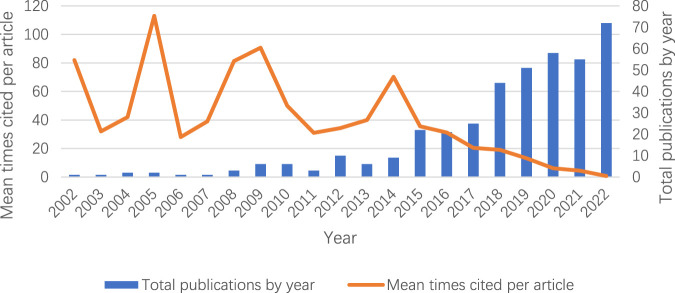
Total publications and mean times cited per article by year on HIIE in children and adolescents from 2002 to 2022.

The output of papers in a research field directly reflects the scholars’ attention to the field, the theoretical level, the speed of development, and the social acceptability. The annual output of HIIE research in children and adolescents and the average citations per year were plotted against the annual data distribution (Figure 1). As shown from Figure 1, the literature in this field did not show growth until 2015, which also shows that the research in this field has experienced a slow development process, transitioning from being highlighted by academia to becoming a hot topic. The publication of the original literature was methodologically rigorous, which also echoed the development and evolution of general disciplines. It is further divided into three stages: (1) from 2002 to 2009, the initial stage of research, during which the annual publication volume remained below 3, and related research did not receive extensive attention. (2) There was slight growth in publications between 2010 and 2014. Although the number of papers published has indicated that the research has lost some momentum, it has guaranteed the quality of research in this field to a certain extent. From a theoretical point of view, this also indicates that there may be new paradigms in the future, such as breakthroughs in methods or theories. (3) From 2015 to 2022, the number of published papers has linearly increased during the rapid development stage of research. During this period, HIIE for health promotion in children and adolescents has become a hotspot in this academic discipline, which may align with the World Health Organization’s appeal and pioneering research institutions and scholars’ contributions to the field. Regarding literature citations, the mean times cited per article ranged from 82 in 2002 to 0.7 in 2022. An article may get enough citations in the field after many years of publication (Ellegaard and Wallin, 2015). Moreover, most of the articles in our dataset are original articles. Compared with some review or report articles, it is recognized that the citations are not high (Joshi, 2014).

Based on the 2002–2022 database, a total of 1723 authors contributed 399 articles (derived from 144 literature sources), including 348 original research articles and 51 reviews, six single-author articles, and the rest were multi-author collaborations. The average number of authors per paper was 4.3, the database contains a total of 1037 keywords, 11957 references, and the average number of citations per year per document is 2.9. Table 1 lists the top 10 prolific authors in this field, the number of papers they contributed, the citations of their research results, and their affiliations and countries. The top three authors with the most published papers are Williams CA, Barker AR, and Lubans DR, who have published 23, 20, and 15 papers, respectively. Lubans DR and Eather N are the most cited authors, both with 200 local citations, and Plotnikoff RC and Costigan SA rank second and third in local citations with 174 and 165 citations, respectively. The number of articles counted comes from datasets retrieved through strict restrictions, so the number of articles will vary through other datasets from PubMed, WoSCC, or Google Scholar. Accordingly, considering that the number of global citations may reduce the relevance of documents and cause difficulties in analysis and interpretation, the local citation score was chosen when calculating the citation frequency of papers. It is the total number of times the article is cited by other articles within the retrieved dataset, i.e., derived from part of the WoSCC data. So, the local citations given by the Bibliometrix R-Package are much lower than the global citations. To further examine the contributions of scholars in this field, the H-index, a quantitative indicator that characterizes the scientific output of researchers, was used. With an unbiased method, the degree of the cumulative contribution of scientists is compared (Hirsch, 2005). The H-index in this study also corroborates the contribution of the relevant authors to the field. Similarly, because the constraints were set when retrieving the data, the H-index of the top 10 authors listed in this study is much lower than their actual H-index. However, these do not significantly affect the interpretation of their field contributions.

**TABLE 1:** Top 10 most prolific authors and authors’ cited times and H-index on HIIE in children and adolescents.

Author	Total publications	Sum of times cited	H-index	Affiliation	Nation
Williams CA	23	145	12	University of Exeter	UK
Barker AR	20	135	11	University of Exeter	UK
Lubans DR	15	200	8	University of Newcastle	Australia
Eather N	13	200	8	University of Newcastle	Australia
Hillman CH	11	104	8	Northeastern University	United States
Sperlich B	11	72	10	University of Würzburg	Germany
Bond B	10	83	7	University of Exeter	UK
Smith JJ	9	64	5	University of Newcastle	Australia
Baquet G	8	41	6	University of Lille 2	France
Berthoin S	8	41	6	University of Lille 2	France

### Analysis of journals and co-cited journals

Based on the number of published articles in this dataset, the top 10 journals with published research results were counted (Table 2). The 5-year impact factor of the journals was listed because sorting by impact factor allows the inclusion of many journals with fewer publications but with high impact (Garfield, 2006). If only sorting by the number of publications on research topics, it may cause some confusion for other researchers. Evaluating the quality of journals is a very complex task and is not the focus of this discussion. In summary, an overall view of which high-quality journals in HIIE and children’s and adolescents’ thematic research have more publications is provided. It is highly recommended that the impact factor of the journals be considered holistically (Table 2) because it is related to the quality of papers and the difficulty of publishing papers required by the journals, which may help researchers focus on relevant journals and submit research results.

**TABLE 2:** Top 10 journals on HIIE in children and adolescents.

Source	Impact factor	Articles
International Journal of Environmental Research and Public Health	4.8	33
Pediatric Exercise Science	2.4	19
Journal of Sports Sciences	4.2	15
Frontiers in Physiology	5.3	14
International Journal of Sports Medicine	3.1	13
Journal of Strength and Conditioning Research	4.5	10
Applied Physiology Nutrition and Metabolism	4.2	9
European Journal of Applied Physiology	3.6	9
International Journal of Sports Physiology and Performance	5.1	9
European Journal of Sport Science	4.1	7

**FIGURE 2:**
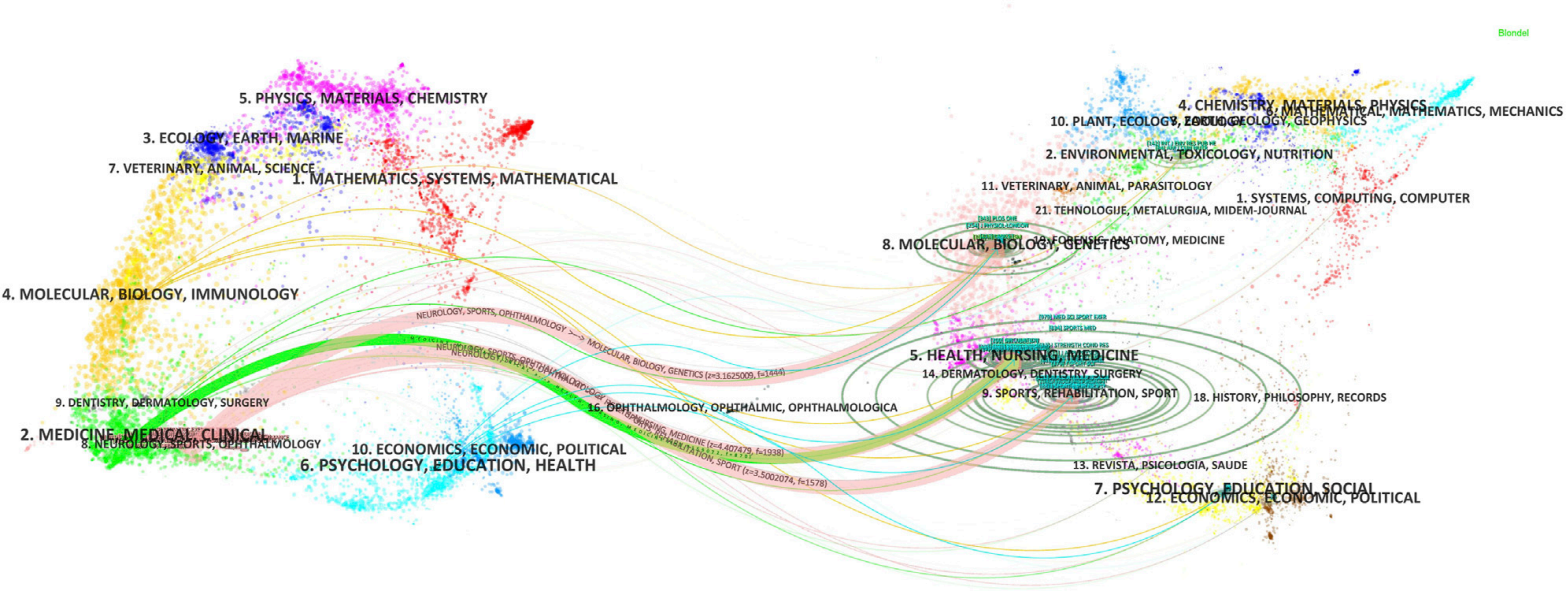
Dual-map overlay of journals that published documents on HIIE research in children and adolescents.

The dual-map overlay of the science mapping literature represents the entire dataset in the context of a global map of science generated from over 10,000 journals indexed in the Web of Science (Chen and Leydesdorff, 2014). The dual-map overlay highlights the distribution of journals in scientific fields and the knowledge flow between citing and cited documents (Li and Chen, 2017). The study analyzes and displays information such as the distribution, citation trajectories, and the center of gravity drift of HIIE research on children and adolescents using a dual-map overlay (Figure 2). The study merged the connections by setting the Z-score to highlight the citing and cited trajectories. The numbers of the double map overlay are the cluster label serial numbers. The label is extracted from the names of journals in the cluster using the log-likelihood ratio (LLR) algorithm. The left side is the citing diagram, the right side is the cited diagram, and the curve is the citation curve, which is used to show the ins and outs of the citation. It was found that the second, eighth, and fourth clusters, including journals focusing on medicine, medical, clinical, neurology, sports, ophthalmology, molecular, biology, and immunology are the most representative sources of citing documents. However, the fifth, eighth, and ninth clusters, including journals focusing on health, nursing, medicine, molecular, biology, genetics, rehabilitation, and sports, are the most representative sources of cited documents. The citing and cited trajectories on the dual-map overlay indicate that HIIE research in children and adolescents has a multidisciplinary trend.

### Analysis of the top cited documents and features

Table 3 lists the top 10 most frequently cited documents based on total citations. The assessment of adolescent health benefits by Costigan et al. (2015) is the most cited literature in the field of HIIE and children and adolescent health promotion, and the evidence on the relative efficacy of adolescent metabolic health by Logan and colleagues is the second most cited paper (Logan et al., 2014). Tjonna et al. was the third most cited study comparing the effects of a multidisciplinary approach and aerobic interval training on cardiovascular risk factors in overweight adolescents (Tjonna et al., 2009). Each of these three articles has been cited more than 35 times. Although there has been disagreement in the academic community on the importance of citations, there are many factors that affect citations, including paper quality, journal impact factor, the number of co-authors on a paper, and institutional and national collaboration, which are significant predictors of citation frequency (Tahamtan, 2016). However, the number of citations is still the most commonly used indicator. At the same time, this study found that 5 of the 10 most cited papers were review articles. A literature review can synthesize past research results, effectively use the valuable experience and professional knowledge base accumulated in this field, and maintain professional judgment and professional practice, playing an increasingly important role in providing evidence-based insights.

**TABLE 3:** Top 10 publications by citation count on HIIE in children and adolescents.

Rank	Title	Journal	Year	Citation count
1	High-intensity interval training for improving health-related fitness in adolescents: a systematic review and meta-analysis	British Journal of Sports Medicine	2015	94
2	High-intensity interval training interventions in children and adolescents: a systematic review	Sports Medicine	2017	51
3	A review of adolescent high-intensity interval training	Sports Medicine	2014	46
4	Aerobic interval training reduces cardiovascular risk factors more than a multi-treatment approach in overweight adolescents	Clinical Science	2009	45
5	Effects of high vs. moderate exercise intensity during interval training on lipids and adiponectin levels in obese young females	European Journal of Applied Physiology	2013	45
6	Greater effects of high- compared with moderate-intensity interval training on cardio-metabolic variables, blood leptin concentration, and ratings of perceived exertion in obese adolescent females	Biology of Sport	2016	35
7	Is high-intensity interval training more effective on improving cardiometabolic risk and aerobic capacity than other forms of exercise in overweight and obese youth? A meta-analysis	Obesity reviews	2016	34
8	Similar health benefits of endurance and high-intensity interval training in obese children	PLOS ONE	2012	28
9	Effect of novel, school-based high-intensity interval training (HIT) on cardiometabolic health in adolescents: Project FFAB (fun fast activity blasts)–an exploratory controlled before-and-after trial	PLOS ONE	2016	27
10	High-intensity interval training on cognitive and mental health in adolescents	Medicine & Science in Sports & Exercise	2016	26

## Collaboration networks for research on HIIE in children and adolescents

**FIGURE 3:**
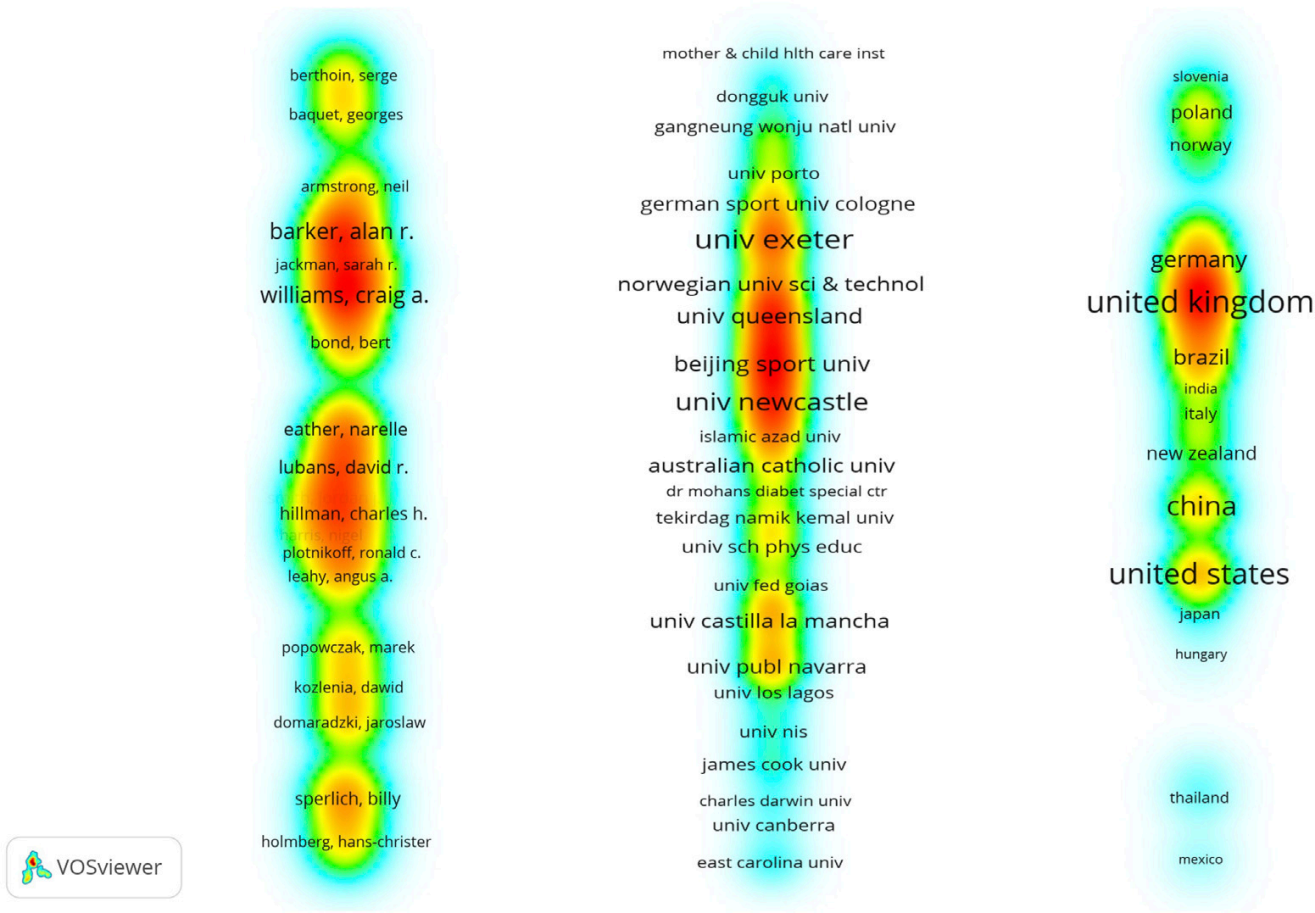
Authors, affiliations, and countries of co-authorship density visualization on HIIE in children and adolescents.

In bibliometric research, there is currently no widely accepted technique that covers all research purposes. VOSviewer is a representative mapping technology for constructing distance-based maps. Since VOSviewer can construct any two-dimensional distance-based map regardless of the mapping technology, this is useful for exploring collaboration overviews such as authors, affiliations, and countries and examining the most critical areas in the map (Van Eck and Waltman, 2010). Therefore, VOSviewer was used to analyze the collaboration network of authors, affiliations, and countries (Figure 3). From Figure 3, we can observe that more significant sub-networks in the collaboration network can be determined. The sub-networks here are generated according to the relatedness of their co-authorship links. A link here is a connection or relationship between two items, and each link has a strength, generally represented by a positive value. The higher the value, the stronger the link. For example, in the case of co-author links, the strength of the link represents the number of publications co-authored by two researchers. This study used Pajek to modify the original random density view into a linear density view in the author collaboration network. Furthermore, some labels may not be displayed in the country collaboration network (i.e., Northeastern University, University Western Australia, Australia, and Spain). VOSviewer automatically presents this to avoid overlapping labels. In the density view, the larger the item label, the more critical it is within the collaboration area. Among other aspects, the color of a node in the visual map is determined by the density of items at that node. The above process requires two steps. In the first step, the density of items at that node is converted to a color value. The higher the item density, the higher the color value. In the second step, the node’s color is determined by matching the color value of the node with the color value in the density color file. To make the critical collaborator network more conspicuous, the color range was set to white–blue–green–red in this research. The larger the number of items about a node, the smaller the distance between the item and the node of interest, and the higher the weight of the adjacent items, the closer the color of the node is to red.

### Analysis of authors in co-authorship

The results of the density visualization analysis of the authors (Figure 3) show that there are 31 projects, 8 cooperative networks, and 85 links in total in the visual map, and the total link strength is 393. The five significant sub-networks are as follows: the research network as represented by American and Australian scholars Hillman and Lubans. Hillman currently focuses on the improvement of cognitive function, cardiorespiratory fitness, working memory, and inhibitory control through physical activity and exercise training, as well as the relationship between aerobic fitness and children’s selective attention. Lubans currently focuses on physical activity intervention in youth, children, and adolescents, with an emphasis on cardiometabolic and mental health, as well as school-based training program optimization. However, their shared interest is also in examining the cognitive benefits of HIIE interventions in adolescents, especially the link between changes in cardiorespiratory fitness, muscle fitness, and cognitive control. Related research has provided important information for public policy. The research network is as represented by British scholars Williams and Barker. The current main research interests of the team include exercise in children with cystic fibrosis, exercise intervention in children with congenital heart disease, adolescents’ physical activity and cardiometabolic health, the effect of different exercise patterns on bone mass in adolescents, and nutritional interventions for children with cancer. In the search subject and time frame, the team mainly explored the effect of HIIE on insulin sensitivity, vascular endothelial function, and heart rate variability, which extended the work compared to the traditional research topics of cardiovascular disease (CVD) risk factors. Related research provides evidence for the feasibility and sustainability of school-based HIIE important reference. The research network is as represented by French scholars Berthoin and Baquet. The team’s current main research interests include children’s physical activity patterns and assessment, children’s sports performance and pulmonary oxygen uptake kinetics, and children and adolescents’ sports responses and ratings, exercise interventions and mechanisms of blood metabolism regulation in patients with type 1 diabetes, non-contact injury mechanisms, and post-exercise recovery. In the search subject and time frame, the team mainly examined changes in the cardiorespiratory function of children and adolescents after HIIE intervention, by improving aerobic fitness, body composition, and cardiometabolic risk in obese adolescents, and reported athletes’ evidence of blood oxygen saturation. The research network is as represented by German and Swedish scholars Sperlich and Holmberg, respectively. The teams investigate tissue saturation index, oxygen consumption, and other parameters of skeletal muscle and exercise performance, as well as the effect of functional strength training on muscle volume, strength, and mobility, along with the athlete’s training intensity, volume, and recovery distribution. In the search subject and time frame, the community mainly studied the effects of HIIE on physiologically relevant parameters, circulatory and metabolic responses, and the intervention effect of HIIE on obese children. Thus, the teams’ research contributes extensive physiological evidence of exercise performance. The research network is as represented by the Polish scholars Domaradzki and Popowczak. The teams currently focus on the effects of changes in anthropometric measurements, physical activity, agility abilities, and myopia, as well as musculoskeletal injury risk in youth and elite players, e.g., to assess the functional form of the relationship between stature, BMI, and change in direction speed and agility and verify the association of myopia with BMI in explaining the formation of functional forms between both balance control and physical activity. In the retrieval subject and time frame, the team mainly explored the prognostic potential of gender, biological age, and body composition in predicting positive changes in cardiorespiratory fitness and cardiovascular parameters after HIIE in adolescents. The teams’ findings contribute to evidence of anthropometric measurements predicting the effects of HIIE intervention. Considering that HIIE has been followed by the top research teams of scientists and the extensive research scope of these research groups, it can be predicted that these topics will continue to be explored.

### Analysis of affiliations in co-authorship

Affiliation’s density visualization analysis results shown in Figure 3 represent a total of 486 items, 31 clusters, and 1755 links in the visual map, with a total link strength of 1976. Among them, the representative top 5 most significant affiliations are the University of Exeter, which contains 28 documents, a total of 422 citations, and a total link strength of 38. The University of Newcastle contains 18 documents, with a total of 456 citations and a total link strength of 46. The Northeastern University contains 12 documents with a total citation count of 245 and a total link strength of 38. University Western Australia contains 10 documents, total citations of 191, and total link strength of 30. The University of Queensland contains 10 documents, 336 total citations, and 40 total link strengths. The top 10 affiliations in terms of publication volume were further analyzed, and it was found that there are three affiliations in the United Kingdom and Australia separately, accounting for 60% of the total. The United States, New Zealand, Spain, and China each have one institution. It can be seen from the above that research affiliations in the UK and Australia have certain research advantages in this field.

### Analysis of countries in co-authorship

The country density visualization analysis results (Figure 3) show that there are 42 items, 9 clusters, and a total of 178 links in the visual map, and the total link strength is 371. Among them, the representative top 5 most significant countries are the United Kingdom, which contains 72 documents, with a total citation count of 1176 and a total link strength of 58; the United States, which contains 65 documents, with a total citation count of 1118 and a total link strength of 73; Australia, which contains 59 documents, with a total citation count of 1447 and a total link strength of 62; China, which contains 51 documents, with a total citation count of 374 and a total link strength of 22; and Spain, which contains 39 documents, with a total citation count of 533 and a total link strength of 43. From the perspective of the total link strength between countries, it is easy to find that the United Kingdom has the highest volume of publications, and the United States has accomplished the best in international cooperation. It is suggested that the above two factors should be comprehensively considered in future cooperation between countries.

### Three-field plot analysis of affiliation, sources, and authors

**FIGURE 4:**
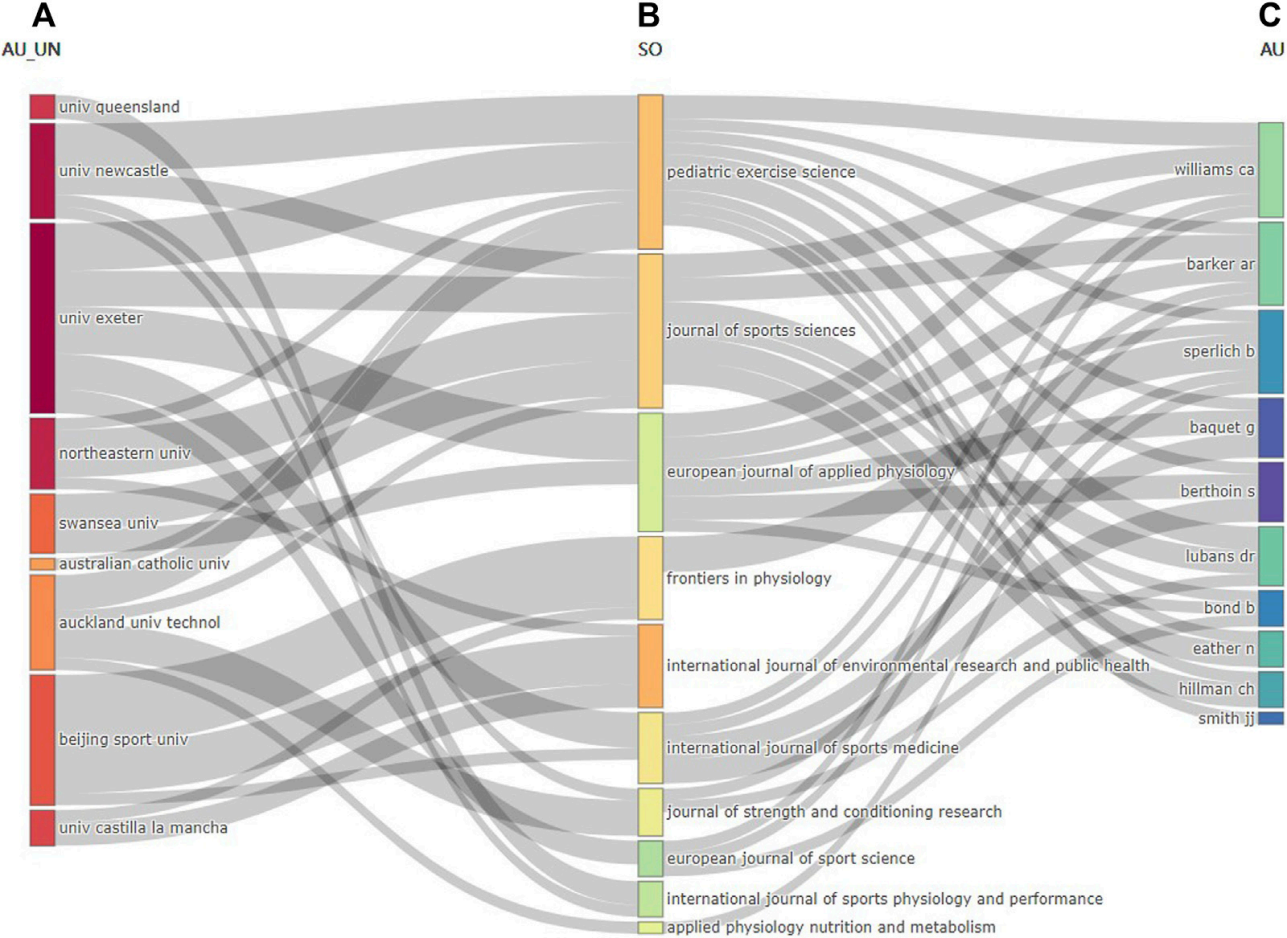
Three-field plot showing the network between affiliations **(A)**, sources **(B)**, and authors **(C)** of outcome outputs on HIIE in children and adolescents from 2002 to 2022.

To further examine the status and preferences for publication in reputable journals by top affiliations and top researchers on HIIE in children and adolescents, the aim is to provide insights for other practitioners in the field and valuable lessons. Based on the retrieved literature, it is set with the top 10 most relevant affiliations on the left, the top 10 most relevant sources in the middle, and the top 10 most relevant authors on the right (Figure 4). Its relationship is visually analyzed. The three data elements have different sizes of rectangles linked by gray lines. The height of the rectangle nodes is proportional to the frequency of the occurrence of a certain affiliation, source, or author within the collaboration network. The width of the lines between the nodes is proportional to the number of connections. The three-field plot based on a Sankey diagram that depicts the connections from affiliations to sources and authors is displayed in Figure 4. The focus of the analysis in the middle is the source, which connects the affiliation and author. Pediatric Exercise Science and the Journal of Sports Sciences are the most significant nodes in both incoming and outgoing flow counts (the count is 13). The most relevant affiliations and authors have been published in this journal, followed by the European Journal of Applied Physiology, Frontiers in Physiology, and the International Journal of Environmental Research and Public Health. The University of Exeter is an affiliation with a significant contribution to publications with an outgoing flow count correlated with almost all journals (the count is 16), followed by Beijing Sport University (the count is 11), and then the University of Newcastle and Auckland University of Technology tied for third place (the count is 8). The top three most relevant authors, Williams CA, Barker AR, and Sperlich B, each have five incoming flow counts correlated with the 10 most relevant journals. Furthermore, their research findings were published more recently in Pediatric Exercise Science, Journal of Sports Sciences, European Journal of Applied Physiology, and Frontiers in Physiology in this dataset.

## Research hotspots of HIIE in children and adolescents with the keyword burst analysis

### Keyword co-occurrence analysis

**FIGURE 5:**
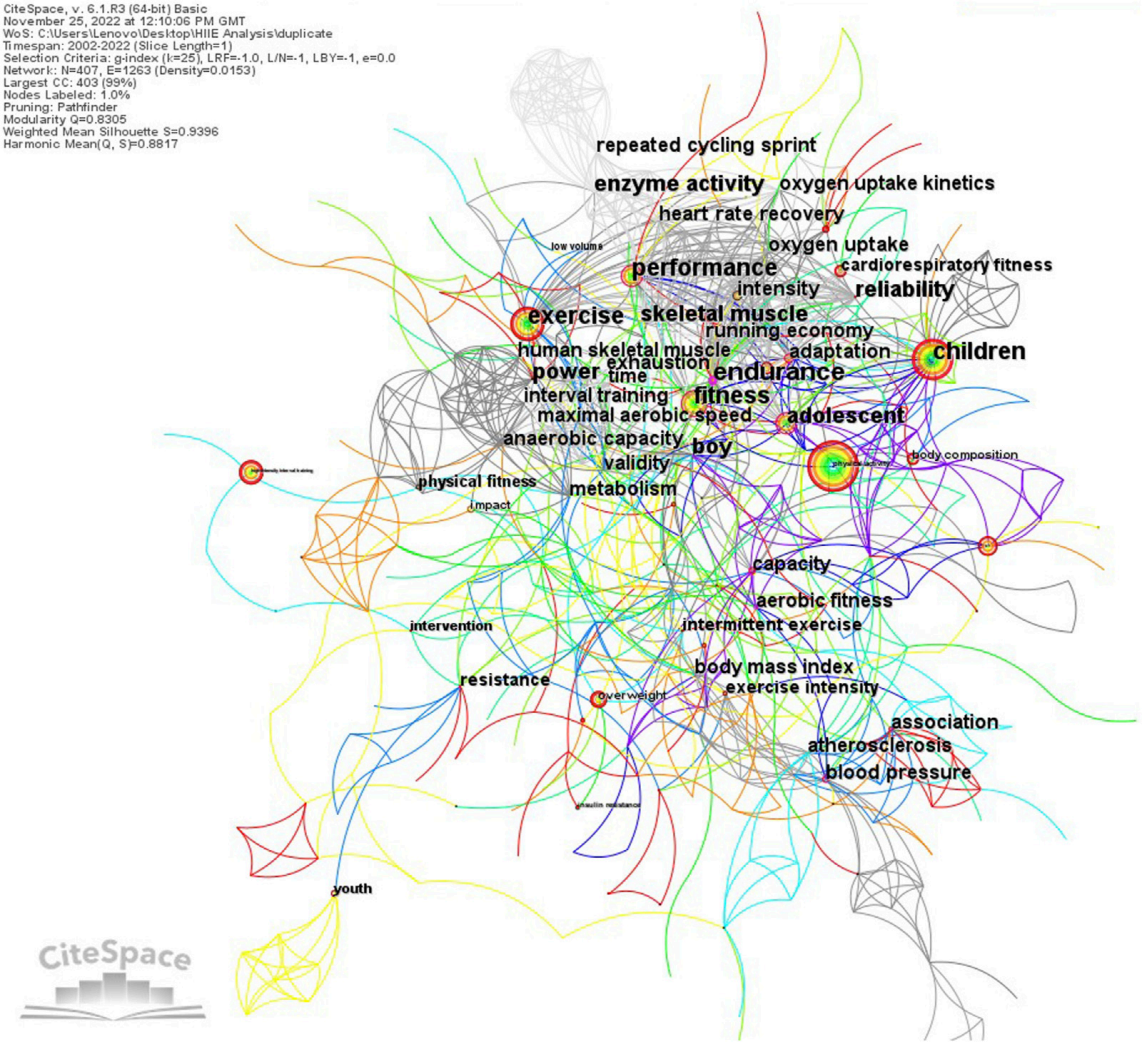
Map of co-occurrence keywords on HIIE in children and adolescents. In the co-occurrence views of various items generated by CiteSpace, the size of nodes represents the frequency of item occurrences, and the connections between nodes reflect the strength of the relationship between items. The full details of the color and the circle layer of the keyword node will be interpreted in the text.

The basic principle of keyword co-occurrence analysis is to count the number of times a set of keywords appears in the same set of documents and use these co-occurrence times to measure the relationship between them. Using CiteSpace to conduct keyword co-occurrence analysis on HIIE research in children and adolescents, the CiteSpace keyword co-occurrence map (Figure 5) illustrates that the larger the node, the higher the frequency of the keyword, and the color of the keyword node varies from cool to warm. The change represents the time from past to present, the circle layer of the keyword node represents the tree ring, the purple circle layer is the centrality (centrality >0.1) that is highlighted, and the width of the tree ring can refer to the size of the centrality. To reflect the influence of keywords, CiteSpace provides a valuable perspective for studying the evolution of scientific networks with an incremental approach, and specific examples of network evolution can lead to insights into broader networks. It uses the thematic river metaphor to describe temporal changes in word frequency. Enhanced themes can be identified if broader word frequency streams can be detected (Chen, 2004). This identification is also why the node size is important to represent the topic. In this study, keyword co-occurrence analysis obtained 407 nodes and 1995 links. CiteSpace keyword co-occurrence analysis should also calculate the betweenness centrality of nodes. Betweenness centrality mainly measures the degree to which a point is located between other points in the network (Freeman, 1978; Brandes, 2001). The importance of this betweenness centrality concept is that it may become a structural hub that controls the flow of information in the network, can facilitate or hinder the transfer of biased information, and has the potential to tie the network together by coordinating the activities of other points (Freeman, 1977). A high betweenness centrality value can identify potentially revolutionary information. Depending on the type of analysis node, it may be informative, e.g., authors, keywords, or documents. For example, an author node will have a very high betweenness centrality if it provides the only connection between two large but otherwise unrelated clusters (Chen, 2005). Therefore, nodes with high betweenness centrality often represent vital nodes in the network, and Table 4 lists the keyword information about the top 20 high betweenness centrality.

**TABLE 4:** Top 20 co-occurrence keywords on HIIE in children and adolescents.

Count	Centrality	Year	Keyword	Count	Centrality	Year	Keyword
31	0.27	2005	Endurance	4	0.13	2009	Atherosclerosis
121	0.20	2005	Children	68	0.12	2005	Performance
67	0.18	2002	Fitness	10	0.12	2013	Arterial stiffness
28	0.18	2009	Blood pressure	6	0.12	2009	Body mass index
19	0.15	2013	Aerobic fitness	5	0.12	2014	Academic achievement
19	0.15	2009	Capacity	57	0.11	2007	Adolescent
13	0.15	2002	Boy	104	0.10	2005	Exercise
33	0.14	2005	Adaptation	19	0.10	2015	Association
16	0.14	2015	Resistance	13	0.10	2002	Power
15	0.14	2004	Skeletal muscle	10	0.10	2009	Validity

### Evolution of keyword clusters

**FIGURE 6:**
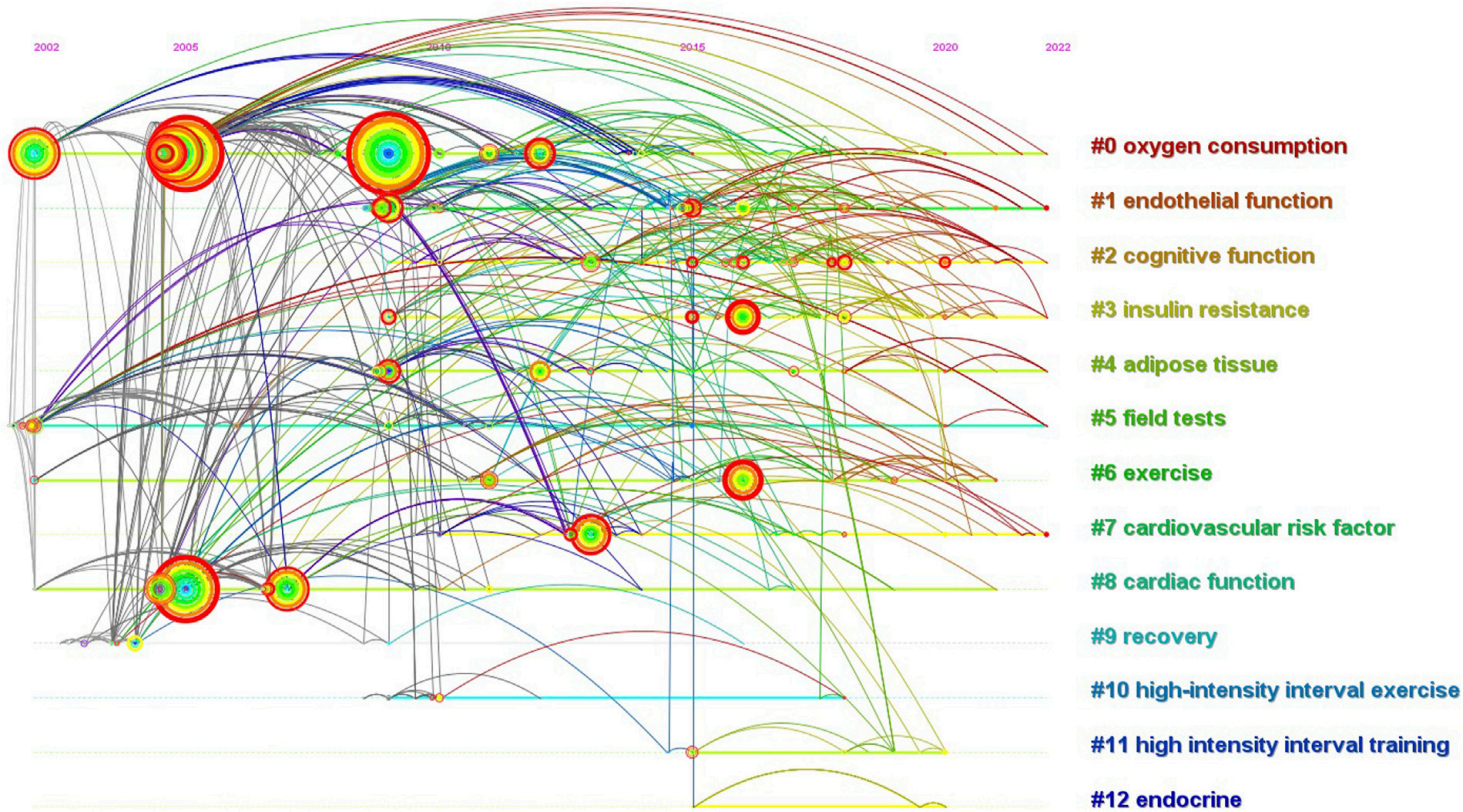
Timeline visualization with overlays of keyword clustering on HIIE in children and adolescents from 2002 to 2022. The X axis of the timeline view is the year, and the Y axis is the cluster label. The view shows the time span and research progress of each cluster.

A visual timeline map was drawn by CiteSpace keyword cluster analysis (Figure 6). The timeline map shows that HIIE has a clear boundary between themes in children and adolescent research, and the evolution path is prominent. Regarding clustering parameters, modularity Q is 0.71, which is used as an evaluation index for network modularity and Q > 0.3 indicates that the network cluster is good. In terms of silhouette, the weighted mean silhouette is 0.88 in this study, S > 0.7, and higher silhouette values indicate higher network homogeneity. Data analysis shows that the clustering structure is significant and clustering is convincing. Figure 6 displays keyword co-occurrence timeline visualization as an overlay. Each cluster is displayed horizontally and progresses from left to right over time. Years per slice are 1, and the node is proportional to the frequency of the keyword. Large-sized nodes or nodes with purple tree rings are of particular interest because they are either high-frequency words, high betweenness centrality words, or both. Colors range from dark to light, denoting themes from earlier to most recent years, with brighter colors representing newer research themes. So, the dominant colors of the clusters were revealed when those themes flourished. The keyword timeline view helped us identify 13 significant clusters. Clusters are numbered from 0, i.e., Cluster#0 is the largest cluster, and Cluster#1 is the second cluster. As shown in the timeline overview, the duration of clusters varies by research topic, with some clusters spanning more than 20 years. In contrast, some clusters are relatively short-lived, such as Cluster#12 endocrine, which has the shortest cluster duration but also spans 5 years. This study used the link walkthrough function of CiteSpace to detect the evolution of each cluster in the timeline view and found that the first references of Cluster#0, Cluster#5, and Cluster#6 appeared in 2002. However, the rate of evolution and development of each cluster are different. For instance, Cluster#0 focusing on oxygen consumption, Cluster#5 on field tests, and Cluster#6 on exercise experienced a decline in vitality in 2009, 2011, and 2016, respectively. Cluster#6 has ended, while relevant topic research on Cluster#0 and Cluster#5 continues within the data’s retrieval range. Conversely, Cluster#0 focusing on oxygen consumption, Cluster#1 on endothelial function, Cluster#2 on cognitive function, Cluster#3 on insulin resistance, Cluster#4 on adipose tissue, Cluster#7 on cardiovascular risk factor, and Cluster#8 on cardiac function are interconnected and affect each other. They are also the research categories that researchers in this field are keen to pay attention to.

### Keyword burst analysis

Keyword burst further visualizes and analyzes current emerging topics in the field. According to Kleinberg (2003), the appearance of topics in a document stream is signaled by “bursts of activity,” with certain features rising sharply in frequency as the topic emerges. The data have a strong temporal characteristic, punctuated by the sharp and sudden onset of particular episodes, and can be organized around rising and falling patterns of activity. In many cases, it can reveal more than people realize, representing the level of research activity or emerging trends. Based on the keyword co-occurrence analysis, a keyword burst analysis was conducted to detect the research hotspots of HIIE in children and adolescents. Twenty high-burst keywords were retrieved through CiteSpace, sorted by the beginning year of burst (Table 5); the data in the chart were directly derived from the CiteSpace keyword burst detection analysis. The time span is from 2002 to 2022, and the strength denotes the citation burst strength; the thin dashed blue lines denote either no burst or the end of burst; and the position and length of the thick dashed red lines denote the start and end time of the burst and the duration of the burst. From this table, it can be found that the keyword with the greatest burst strength, e.g., risk with burst strength, is 3.70, which means that risk may be a turning point in the entire network. The keyword with the longest emergence time is boy, which has been emerging from 2002 to 2017, meaning that the topic has longevity as a research theme. It is worth noting that in this dataset, the span of the retrieved literature is 2002–2022, which indicates that boys and interval training may also have been research hotspots before 2002. The keyword that continued to burst when the literature was searched was executive function, which is a novel finding from this pediatric field. However, the statistics show that the topics of cognitive performance and high-intensity interval training are current research hotspots. This is not a simple repetition. In the context of many unsolved problems, such as the dose–response relationship of HIIE and the optimization of the protocol, HIIE research still has a long way to go. In particular, the current education field has not made HIIE a top priority in the classroom. Although educational leaders have addressed seemingly more critical issues such as academic achievement, there is a lack of sufficient scientific understanding of adolescent health. This oversight ignores chronic disease risk and the hidden dangers that may take more time to fix. It is speculated that when the relevant theory of HIIE is mature enough, this problem will be taken more seriously. In addition, it was found that although keyword bursts have ended within 5 years of the data retrieval deadline, e.g., insulin resistance, heart rate variability, and high-intensity interval training, the problems may not have been resolved. This means that new research designs, methods, and theories related to them may appear in the future, and further elaboration on them in research frontiers may be required.

**TABLE 5:** Top 20 keywords with strongest citation bursts on HIIE in children and adolescents.

Keyword	Year	Strength	Begin	End	2002–2022
Boy	2002	3.54	2002	2017	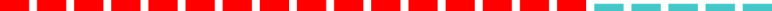
Interval training	2002	2.19	2002	2016	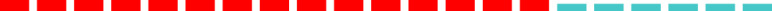
Enzyme activity	2002	2.27	2003	2013	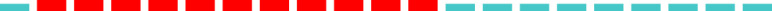
Human	2002	3.08	2009	2012	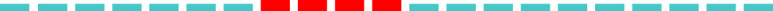
Intermittent exercise	2002	2.91	2009	2012	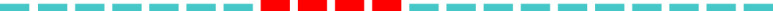
Validity	2002	2.53	2009	2015	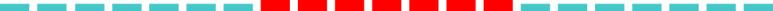
Sprint interval	2002	3.49	2010	2012	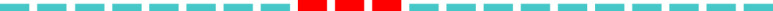
Cardiovascular disease	2002	2.43	2010	2017	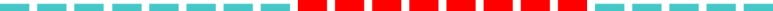
Metabolism	2002	3.70	2011	2015	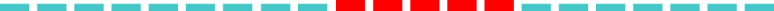
Skeletal muscle	2002	2.46	2014	2016	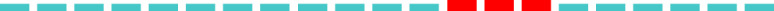
Risk	2002	3.70	2015	2016	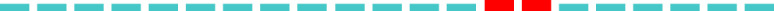
Interval	2002	2.91	2015	2016	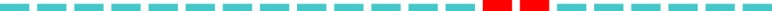
Meta-analysis	2002	3.46	2016	2018	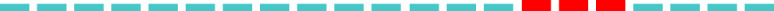
Fitness	2002	2.90	2016	2017	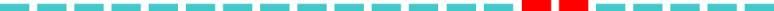
Childhood obesity	2002	2.96	2017	2019	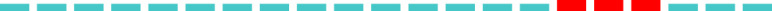
Obesity	2002	2.78	2017	2018	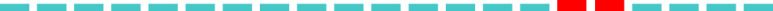
Insulin resistance	2002	3.14	2018	2020	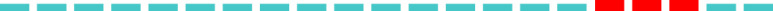
Heart rate variability	2002	3.02	2018	2019	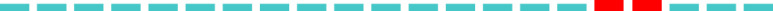
High-intensity interval training	2002	2.26	2018	2020	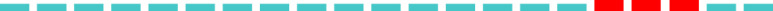
Executive function	2002	3.39	2020	2022	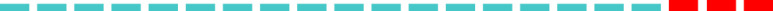

## Analysis of HIIE research frontiers based on the document co-citation analysis

Henry Small first proposed document co-citation analysis in 1973, and it is believed that co-citation clustering provides a new way to study the scientific professional structure (Small, 1973). Co-citation is defined as the frequency with which two scientific articles are cited simultaneously. For example, when a group of authors cites a standard set of articles, these co-citations indicate that these articles may contain conceptual symbols: an idea recognized by peers, an experiment, or a method (Trujillo and Long, 2018). The pattern of bibliographic references indicates the nature of the scientific research frontier, and it is concluded that journal citations provide the most readily available data for measuring the degree of strategic centrality in maps of the scientific literature (Price, 1965). A research frontier represents the state-of-the-art thinking of a research field. Moreover, the research frontier is an emergent and transient grouping of concepts and underlying research issues. The intellectual base of a research frontier is its citation and co-citation footprint in the scientific literature (Chen, 2006). So, the citing articles represent a research frontier, and the cited articles constitute an intellectual base (Persson, 1994).

**FIGURE 7:**
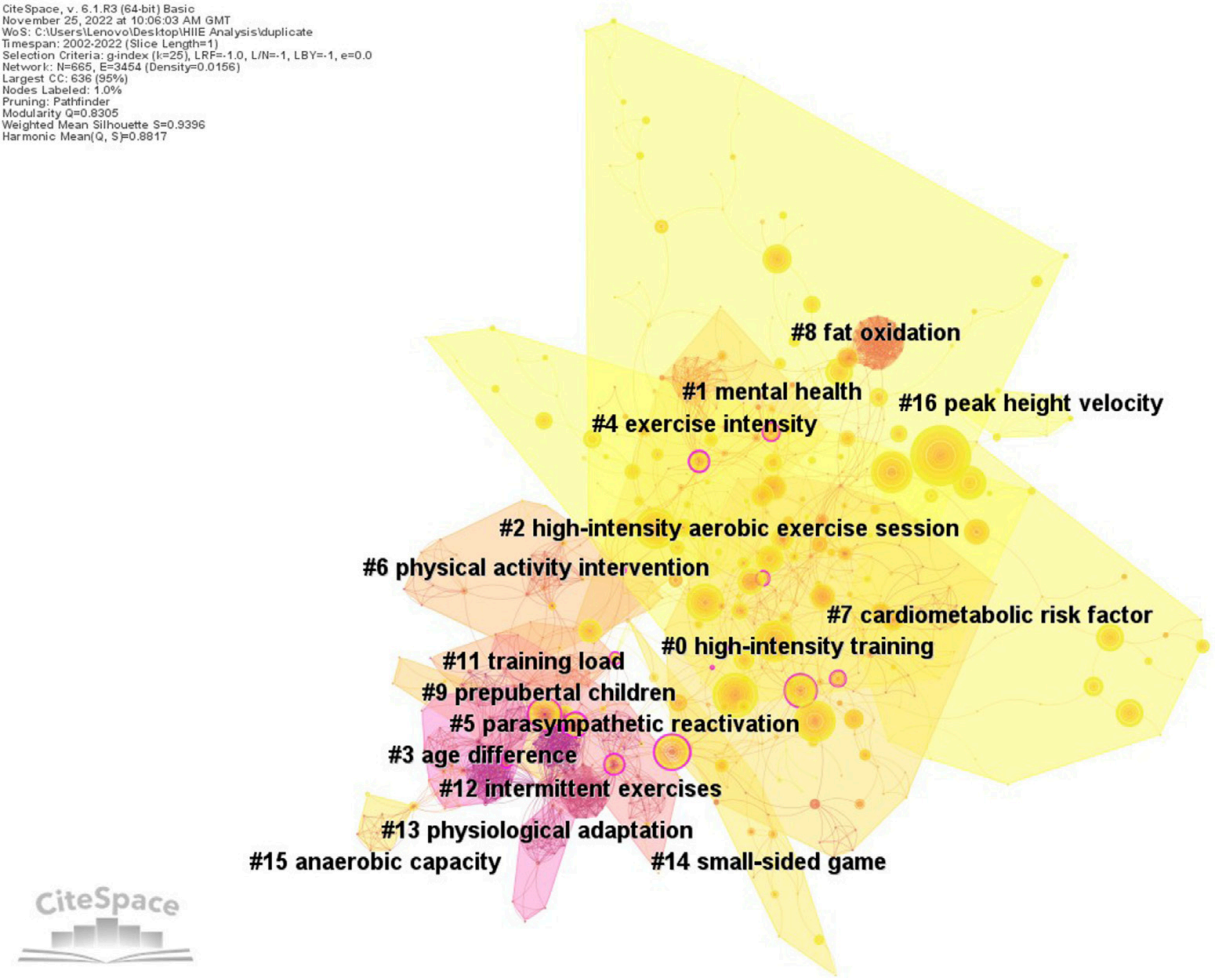
Visualization network and clustering of document co-citation analysis on HIIE in children and adolescents.

Figure 7 shows the co-citation network of the literature on HIIE research, and the clusters and features were accurately identified through CiteSpace. This clustering uses topics to extract tags and LLR algorithms. CiteSpace offers three types of cluster-labeling extraction algorithms: inverted document frequency, LLR, and mutual information (Chen, 2010). LLR is utilized to present how often, or more likely, one model than the other model covers the data, usually providing the best results in terms of uniqueness and coverage of topics associated with clustering. The generative network contains 665 nodes of documents cited and 4198 co-citation links. The color of the color blocks in the cluster corresponds to the average year of each node within the cluster. Regarding network evaluation indicators, modularity Q is 0.83 and weighted mean silhouette S is 0.94. The modularity Q measures the extent to which a network can be divided into independent blocks; a low modularity suggests a network that cannot be reduced to clusters with clear boundaries, whereas a high modularity may imply a well-structured network (Chen et al., 2010). The silhouette value is a parameter used to evaluate the relative quality of clusters based on comparing their tightness and separation; the larger the silhouette value of a network, the higher the network homogeneity (Rousseeuw, 1987). The higher the average score of silhouettes, the more influential a group is in terms of clusters.

The CiteSpace document co-citation cluster analysis revealed a total of 16 clusters. However, caution must be raised that although the early and mid-term clusters may synthesize knowledge from different fields, there is a clear sign of cross-border links throughout the network (Chen, 2012). However, document co-citation analysis did not find a larger burst, which means that these clustering topics have become outdated or solved. Thus, they are not the research frontiers this article is trying to discover, but they exist more as background information. Therefore, this study only analyzed four clusters that provide new information and have a larger burst simultaneously. The four largest document co-citation clusters are high-intensity training (cluster #0), mental health (cluster #1), exercise intensity (cluster #4), and cardiometabolic risk factors (cluster #7). Table 6 shows the five most cited documents in the largest document co-citation cluster, and Table 7 shows the cluster composed of citing documents, which maps the research frontiers in this field. This study analyzed two aspects of each cluster: (a) prominent members of the intellectual basis and (b) themes identified in the citers of the cluster as research frontiers.

**TABLE 6:** Most-cited references in the four largest document co-citation clusters ranked by burst on HIIE in children and adolescents.

Cluster ID	Citation count	Burst	Centrality	Sigma	Cited reference
#0	34	5.75	0.20	2.88	Burgomaster KA et al. (2008); Similar metabolic adaptations during exercise after low-volume sprint interval and traditional endurance training in humans
	9	5.59	0.04	1.25	Wisloff et al. (2007); Superior cardiovascular effect of aerobic interval training versus moderate continuous training in heart failure patients
	18	5.39	0.05	1.31	Sperlich et al. (2011); Effects of 5 weeks of high-intensity interval training vs. volume training in 14-year-old soccer players
	11	5.38	0.06	1.40	Crisp et al. (2012); adding sprints to continuous exercise at the intensity that maximizes fat oxidation: implications for acute energy balance and enjoyment
	26	4.30	0.06	1.29	Corte de Araujo et al. (2012); Similar health benefits of endurance and high-intensity interval training in obese children
#1	12	5.14	0.01	1.04	Slaughter MH et al. (1988); Skinfold equations for the estimation of body fatness in children and youth
	12	4.78	0.05	1.28	Bond et al. (2015); The acute effect of exercise intensity on vascular function in adolescents
	13	4.48	0.07	1.37	Diamond (2013); Executive functions
	18	4.31	0.02	1.07	Leahy et al. (2019); Feasibility and preliminary efficacy of a teacher-facilitated high-intensity interval training intervention for older adolescents
	12	3.13	0.01	1.03	Malik AA et al. (2017); Acute cardiorespiratory, perceptual, and enjoyment responses to high-intensity interval exercise in adolescents
#4	16	5.16	0.12	1.78	Barker AR et al. (2011); Establishing maximal oxygen uptake in young people during a ramp cycle test to exhaustion
	7	3.58	0.04	1.16	Thackray AE et al. (2013); Acute high-intensity interval running reduces postprandial lipemia in boys
	8	3.42	0.01	1.03	Bond B et al. (2015); Exercise intensity and postprandial health outcomes in adolescents
	16	3.33	0.28	2.25	Carson V et al. (2014); Vigorous physical activity and longitudinal associations with cardiometabolic risk factors in youth
	11	3.28	0.06	1.21	Babraj JA et al. (2009); Extremely short-duration high-intensity interval training substantially improves insulin action in young healthy males
#7	24	5.76	0.02	1.11	Dias KA et al. (2018); effect of high-intensity interval training on fitness, fat mass, and cardiometabolic biomarkers in children with obesity: a randomized controlled trial
	22	3.37	0.02	1.06	Weston KS et al. (2014); High-intensity interval training in patients with lifestyle-induced cardiometabolic disease: a systematic review and meta-analysis
	90	0.00	0.01	1.00	Costigan SA et al. (2015); High-intensity interval training for improving health-related fitness in adolescents: a systematic review and meta-analysis
	43	0.00	0.03	1.00	Logan et al. (2014); A review of adolescent high-intensity interval training
	32	0.00	0.01	1.00	Batacan RB et al. (2017); Effects of high-intensity interval training on cardiometabolic health: a systematic review and meta-analysis of intervention studies

**TABLE 7:** Most-citing references in the four largest document co-citation clusters ranked by coverage on HIIE in children and adolescents.

Cluster ID	Size	Silhouette	Label (LLR)	Coverage and citing reference
#0	127	0.907	high-intensity interval training (2007)	34 and Bond B et al. (2015); Two weeks of high-intensity interval training improves novel but not traditional cardiovascular disease risk factors in adolescents
				28 and da Silva et al. (2023); School-based high-intensity interval training programs for promoting physical activity and fitness in adolescents: a systematic review
				20 and Bond B et al. (2015); Accumulating exercise and postprandial health in adolescents
				20 and Domaradzki (2022); Prevalence of positive effects on body fat percentage, cardiovascular parameters, and cardiorespiratory fitness after 10-week high-intensity interval training in adolescents
				19 and Martin-Smith et al. (2020); High-intensity interval training improves cardiorespiratory fitness in healthy, overweight, and obese adolescents: a systematic review and meta-analysis of controlled studies
#1	85	0.908	mental health (2013)	14 and Wassenaar et al. (2021); The effect of a one-year vigorous physical activity intervention on fitness, cognitive performance, and mental health in young adolescents: the fit to study cluster randomized controlled trial
				14 and AI, J et al. (2021); The effect of acute high-intensity interval training on executive function: a systematic review
				11 and Leahy A et al. (2020); Review of high-intensity interval training for cognitive and mental health in youth
				10 and Alves A et al. (2021); High-intensity interval training upon cognitive and psychological outcomes in youth: a systematic review
				8 and Hatch L et al. (2021); Effect of differing durations of high-intensity intermittent activity on cognitive function in adolescents
#4	47	0.959	exercise intensity (2006)	31 and Bond B et al. (2015); Exercise intensity and the protection from postprandial vascular dysfunction in adolescents
				23 and Bond et al. (2015); The acute effect of exercise intensity on vascular function in adolescents
				10 and Bond et al. (2015); Exercise intensity and postprandial health outcomes in adolescents
				7 and Cockcroft et al. (2017); Acute exercise and insulin sensitivity in boys: a time-course study
				5 and Malik A et al. (2019); Perceptual and cardiorespiratory responses to high-intensity interval exercise in adolescents: does work intensity matter?
#7	31	0.980	cardiometabolic risk factors (2012)	8 and Ingul C et al. (2018); Effect of high-intensity interval training on cardiac function in children with obesity: a randomized controlled trial
				8 and Dias K et al. (2018); Effect of high-intensity interval training on fitness, fat mass, and cardiometabolic biomarkers in children with obesity: a randomized controlled trial
				5 and Playsic et al. (2020); Effects of high-intensity interval training and nutrition advice on cardiometabolic markers and aerobic fitness in adolescent girls with obesity
				5 and da Silva et al. (2023); Effects of high-intensity interval training on endothelial function, lipid profile, body composition, and physical fitness in normal-weight and overweight-obese adolescents: a clinical trial
				4 and Faria W et al. (2020); Effects of two methods of combined training on cardiometabolic risk factors in adolescents: a randomized controlled trial

### Cluster#0 high-intensity training

This cluster is the most prominent co-cited cluster of documents in this study. The most cited document discussed HIIE as a “time-efficient” strategy to improve the skeletal muscle oxidative capacity. Previous speculation was confirmed in this study with adults as subjects (Burgomaster et al., 2008). The other four most-cited references focused on cardiovascular function (Wisløff et al., 2007), exercise performance (Sperlich et al., 2011), intensity and fat oxidation (Crisp, Fournier, 2012), and peak VO_2_ (Corte de Araujo et al., 2012). Specifically, the effectiveness of HIIE in improving systolic function and brachial artery flow-mediated dilation in senior patients with chronic heart failure and health-related parameters in obese children was confirmed. Considering the established safety of HIIE, these findings provide an essential reference for future studies on children. According to these cited references, it can be shown that the feasibility and scientific delivery of HIIE in children were initially doubtful, which may be the reason why researchers continued to accumulate further evidence.

The following discussion on the citing literature, i.e., representing research frontiers, may give researchers additional insights into the nature of this cluster. The most-citing references examined the favorable outcome of flow-mediated dilation and heart rate variability (Bond et al., 2015), postprandial systolic blood pressure and resting fat oxidation (Bond et al., 2015), the positive effect of cardiorespiratory fitness (Martin-Smith et al., 2020), and the utility of HIIE programs integrated into physical education lessons (da Silva Bento, Páez and de Mendonça Raimundo, 2021; Domaradzki, 2022). The analysis of the citing literature in this cluster found researchers focused on the time course of metabolic benefits, e.g., that the most metabolic benefit was lost 3 days after the cessation of training, the improvement of cardiorespiratory fitness, and the potential for the introduction of HIIE in the school context. Researchers were mainly concerned about the appropriate exercise intensity and adherence issues because availability of time can be a challenge for children and adolescents to engage in exercise. This observation becomes particularly interesting for those involved in school curricula because low-volume HIIE exercise protocols may provide a pragmatic adjunct to the health benefits of physical education lessons (Martin-Smith et al., 2020; Domaradzki, 2023). However, implementing HIIE as a time-efficient strategy in PE lessons is not yet fully resolved (Delgado-Floody, 2019; Liu, 2024). In this context, high-intensity training in low-volume and school-based settings becomes a frontier research topic.

### Cluster#1 mental health

The most-cited document in the cluster assessed the predictability of body fatness. The equations presented in the study used a multicomponent approach and accounted for the biological immaturity of children, which may provide a more accurate estimate in later relevant studies (Slaughter et al., 1988). Other representative most-cited references focus on the exercise intensity and the time course (Bond, 2015), cardiorespiratory health (Leahy et al., 2019), cardiorespiratory and enjoyment responses (Malik, 2017), and physical activity and cognitive health (Diamond, 2013), principally illustrating that HIIE might provide superior vascular benefits than moderate-intensity continuous training (MICE) and highlighting that HIIE might serve as a strategy to promote health in youth and the importance of physical activity (PA), e.g., lack of exercise impairs executive function. It can be found that not all cited articles focus on mental health, which is logical and acceptable because any methodologies and conclusions can relate to mental health and may be cited by researchers. However, the citation relationship of this cluster presents a new discovery. The vigorous exercise intensity and enjoyment of HIIE are paradoxical when feasibility is concerned. This has drawn physiologists’ and psychologists’ attention to the association between HIIE intervention and the mental health of children and adolescents.

The citing literature analysis of the cluster found that HIIE is used in studies examining the causal relationship between physical activity and cardiorespiratory fitness, cognitive performance, and mental health (Wassenaar et al., 2021), but large amounts of missing data and poor fidelity with the intervention limit the extent to draw conclusions in the study. However, other studies reported that HIIE has positive effects on cognitive performance and psychological outcomes, i.e., executive function (Ai et al., 2021; Leahy et al., 2020), linguistic reasoning, concentration, and selective attention (Alves et al., 2021), in children and adolescent cohort. Furthermore, the dose–response relationship between exercise duration and cognition was discussed in the citing article of this cluster (Hatch et al., 2021). This cluster focuses on the cognitive benefits, but the concerns of the small number of studies and large heterogeneity and the time-course of effects on cognition, following HIIE, are not well-understood. Moreover, in a special setting, e.g., regular short sleep, the consensus on the cognitive performance of exercise and its potential therapeutic benefits remains contested (Saner et al., 2020; Saner et al., 2023; You et al., 2023; You et al., 2023). MICT appears to induce more cognitive benefits than HIIT despite short sleep (You et al., 2023). Sedentary and insufficient sleep among children and adolescents has been a growing concern; these unhealthy lifestyles adversely affect the youth’s mental health (Sampasa-Kanyinga et al., 2020; Gilchrist et al., 2021; You, et al., 2023). Consequently, more high-quality studies focusing on specific protocols are essential to validating existing trends. As noted, this cluster is dominated by experts from other fields who bring new knowledge that the pediatric physiology field does not have, which will mean the emergence of new paradigms in the future. This study anticipates that the theme will continue to be explored due to sustained societal attention to academic performance in children and adolescents worldwide.

### Cluster#4 exercise intensity

The most-cited document in the cluster is Barker’s (2011) formative work about determining peak VO_2_ in young people. This research outlined that supramaximal protocols elicit a peak VO_2_ similar to the ramp protocol, thus satisfying the plateau criterion despite only being present in 30% of the initial ramp responses. As the gold standard for measuring aerobics, a valid peak VO_2_ plays a crucial role when attaining objective results, so other researchers broadly cited this finding. The other four most-cited references focused on postprandial plasma triacylglycerol (Thackray et al., 2013), postprandial risk factors (Bond et al., 2015), cardiometabolic risk factors (Carson et al., 2014), and insulin sensitivity (Babraj et al., 2009) after intensity intervention. The most-cited references generally came to encouraging conclusions, i.e., favorable changes in postprandial SBP, cardiorespiratory fitness, insulin action, and waist circumference after intensity intervention, which deliver valuable references for later research.

In the citing literature of this cluster, researchers focused on exercise intensity improving vascular function after a high-fat meal (Bond et al., 2015), independent effects of exercise intensity on macro- and microvascular functions (Bond et al., 2015), favorable changes in postprandial systolic blood pressure and lipid oxidation (Bond et al., 2015), the time course of adaptations in insulin sensitivity (Cockcroft et al., 2017), and the relationship between different intensities and affective experience and cardiorespiratory responses (Malik et al., 2019). These findings show that performing HIIE may provide superior health outcomes compared to MICE in children and adolescents, e.g., the macrovascular function was improved in the hours after HIIE but not after MICE; both exercise bouts promoted microvascular function, with the magnitude of this increase being greater after HIIE (Bond et al., 2015); favorable changes in postprandial SBP and lipid oxidation are possible after HIIE but not after MICE (Bond et al., 2015). Furthermore, researchers generally support the use of HIIE as an attractive, feasible, and effective strategy to improve relevant health outcomes in adolescents. However, this study finds that most of the citing articles adopt a single-day protocol to detect relevant health outcomes, which may lead to an inadequate interpretation due to the absence of accumulating intensity (Silva et al., 2023; Liu et al., 2024b). Nonetheless, in the context of physical activity interventions designed to improve youth participation and adherence that have not been successful, these cutting-edge studies offer insightful knowledge and impact the practicality of HIIE as a strategy to promote health benefits in this cohort.

### Cluster#7 cardiometabolic risk factors

The most-cited document in the cluster determined the efficacy of HIIE for increasing cardiorespiratory fitness and reducing adiposity in obese children (Dias et al., 2018). This is the first study to compare the efficacy of HIIE, MICE, and nutrition-only interventions on visceral adipose tissue and subcutaneous adipose tissue volume in obese children. The other four most-cited references focused on cardiorespiratory fitness (Costigan et al., 2015; Weston et al., 2014), metabolic health (Logan et al., 2014), and novel markers of CVD risk (Batacan et al., 2017). The most-cited references generally drew positive conclusions, i.e., HIIE can significantly improve cardiorespiratory fitness, BMI, and body fat percentage. However, researchers highlight that the duration of activity undertaken is essential as time efficiency is of key interest regarding HIIE, e.g., short-term HIIE (<12 weeks) significantly improved VO_2max_, diastolic blood pressure, and fasting glucose. Long-term HIIE (≥12 weeks) significantly improved waist circumference, body fat percentage, resting heart rate, and systolic blood pressure, in addition to the above benefits. These studies were conducted in the context of the emerging prevalence of HIIE as an alternative to continuous aerobic exercise, which is deemed a prospective marker of cardiometabolic risk factors.

The analysis of the citing literature found that this clustering focused on the efficacy of HIIE on cardiac function (Ingul et al., 2018), favorable evidence in cardiorespiratory fitness (Dias et al., 2018), positive effects on insulin sensitivity, body fat, and diastolic blood pressure (Playsic et al., 2020), improvement of endothelial function, lipid profile, body composition (da Silva et al., 2020), and reduction in the cardiometabolic risk Z score (Faria et al., 2020). Ingul et al. (2018) reported equally efficacious HIIE and MICE, i.e., both protocols exhibited superior systolic function during peak exercise, following the interventions compared to the nutrition group. Other findings showed that HIIE was generally superior to MICE in terms of favorable health outcomes. However, there was no explicit conclusion in terms of cardiometabolic biomarkers; further research is warranted in pediatric populations to contribute to the paucity of evidence and consideration of protocol development associated with the diet change (Gesek, Fornal and Zarzycka, 2023; Hanssen et al., 2023). Interestingly, four citing literature reports had overweight or obese children and adolescents as subjects in this cluster, and only one literature reported that the BMI of the subjects was in the normal range. However, considering most children or adolescents are asymptomatic individuals who are inactive and have less physical activity may mean the development of cardiometabolic disease in adulthood (Agirbasli, Tanrikulu and Berenson, 2016; Mikola et al., 2017). Therefore, researchers should pay attention to those children and adolescents who appear “normal” (in body mass) in the future. Regardless, the frontiers of software discovery are enlightening because these citing articles attract the attention of scientists. This clustering may become more apparent as new research is published.

## Limitations

First, there is the potential for publication bias. Due to the limitations of statistical tools on the completeness of the document format, only the Web of Science Core Collection database was selected to search the documents and was limited to publications in English. Furthermore, due to the limitation of the bibliography, which extends only up to 2022, the study recommends readers to refer to more recent sources for the latest developments. Second, there is always a specific time lag in CiteSpace’s exploration of research frontiers because a high-quality, innovative article needs a long time from being published to becoming highly cited. So the research frontiers being explored are subject to change. However, in the context of less literature examining the research frontiers in this field, this research provides researchers with a research method that can be used for future reference.

## Conclusion

This paper has shown a systematic method seldom used in exercise science, which is supported by CiteSpace, VOSviewer, Pajek, and Bibliometrix for deeper and quantitative investigation of emerging and interesting topics in pediatric exercise science. In several novel ways, this paper can be considered a demonstration for studying a new field concisely but quickly. In this case, HIIE in children and adolescents was used as an example, which provides a methodological contribution to the published pediatric literature base.

This article reviewed the developmental history of HIIE in children and adolescents, based on 399 publications published between 2002 and 2022. Key authors, affiliations, countries, sources, keywords, and references were identified. Furthermore, predictions based on insulin resistance, heart rate variability, high-intensity interval training, and executive function as research hotspots in recent years for HIIE in children and adolescents were found. The research frontiers of document co-citation clustering mapping show high-intensity training, mental health, exercise intensity, and cardiometabolic risk factors to be prominent. In conclusion, the employment of a combination of quantitative and qualitative approaches to increase the understanding of HIIE evolutionary processes, research hotspots, and development trends was highlighted.
